# Study on the Mechanism of Hearing Loss Induced by USH2A Gene Knockout

**DOI:** 10.1155/humu/8451291

**Published:** 2026-05-28

**Authors:** Chi Chen, Baicheng Xu, Jiong Dang, Huan Tan, Panpan Bian, Yanli Wang, Yufen Guo

**Affiliations:** ^1^ Department of Otolaryngology-Head and Neck Surgery, Lanzhou University Second Hospital, Lanzhou, Gansu, China, ldey.cn

**Keywords:** outer hair cells, transcriptome sequencing, USH2a, Usher syndrome

## Abstract

**Background:**

Usher syndrome (USH) is an autosomal recessive disorder characterized by hearing loss, retinitis pigmentosa, and variable vestibular dysfunction. USH2A is one of the causative genes of USH. This study is aimed at exploring the mechanism of hearing loss induced by USH2A gene knockout.

**Method:**

USH2A knockout (Ush2a^−/−^) mice were used, and auditory brainstem response testing was performed on WT, Ush2a^−/−^, and Ush2a^+/−^ mice. Then, the cochlea tissues were used to carry out immunofluorescence staining, hematoxylin and eosin (H&E) staining, and scanning electron microscopy (SEM). The mRNA expressions were detected by RT‐qPCR. Finally, the differentially expressed genes (DEGs) in cochlear tissues of Ush2a^−/−^ and WT mice were identified by transcriptome sequencing.

**Results:**

Compared to WT mice, Ush2a^−/−^ and Ush2a^+/−^ mice exhibited moderate‐to‐severe nonprogressive hearing loss, with more pronounced deficits at low (4 kHz) and high (32/24 kHz) frequencies. HE staining and immunofluorescence staining showed that the modiolus, stria vascularis, basilar membrane, and the number of inner hair cells and outer hair cells (OHCs) in USH2A knockout mice have not changed. However, SEM results showed that severe stereociliary collapse was evident in OHCs of the Ush2a^−/−^ group. In addition, through transcriptomic analysis, 3632 upregulated genes and 2921 downregulated genes were obtained in the Ush2a^−/−^ mice. Among these DEGs, the most DEGs associated with hearing loss were Scn2a, Shank2, Bsn, Fcer1g, Prkce, Tgfb1, and Irf7.

**Conclusion:**

This study demonstrates that USH2A deficiency disrupts auditory function through stereociliary instability and dysregulation of genes critical for synaptic transmission and cytoskeletal dynamics.

## 1. Introduction

Usher syndrome (USH), also known as hereditary deafness‐retinitis pigmentosa syndrome, is an autosomal recessive disorder characterized by hearing loss, retinitis pigmentosa, and variable vestibular dysfunction, representing the most common genetic condition affecting both auditory and visual systems [1]. The incidence rate of USH is 1/25,000 [2]. Clinically, USH is classified into three subtypes (USH1, USH2, and USH3) based on genotype–phenotype correlations. Among these, USH2 accounts for 56%–67% of cases, with hearing loss typically manifesting between 8 months and 14 years of age, and retinal degeneration emerging between 10 and 38 years [3, 4]. USH2 exhibits genetic heterogeneity, with three causative genes: USH2A, ADGRV1/GPR98, and WHRN/DFNB31, leading to diverse clinical presentations even within the same genotype.

Mutations in USH2A are associated with hereditary hearing loss and retinitis pigmentosa (RP39), though isolated retinal degeneration without hearing impairment has also been reported. ADGRV1/GPR98 mutations are linked to combined auditory and visual deficits, with rare associations to reflex epilepsy [5]. WHRN/DFNB31 mutations cause hearing loss and retinal degeneration, but isolated nonsyndromic hearing loss (DFNB31) can also occur [6]. Despite the clinical significance of USH2A in auditory‐visual disorders, there remains a paucity of studies systematically investigating the correlation between USH2A genotypes, audiometric phenotypes (e.g., severity and configuration of hearing loss), and age‐related progression, particularly in Chinese populations.

To address this gap, we established a USH2A knockout mouse model to investigate the auditory consequences of USH2A gene disruption. Transcriptomic analysis was further employed to identify differentially expressed genes (DEGs) in Ush2a^−/−^ mice, aiming to elucidate the molecular mechanisms underlying USH2A‐related hearing loss and provide insights into genotype–phenotype interactions in USH.

## 2. Materials and Methods

### 2.1. Mouse Genotype Identification

**Figure 1:**
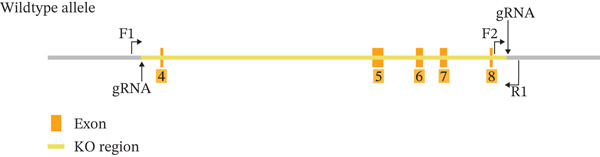
The gene knockout strategy.

Ush2a knockout (Ush2a^−/−^) mice were purchased from Suzhou Saiye Biotechnology Co. Ltd. Briefly, using CRISPR/Cas gene editing technology, Exons 4–8 of the USH2A gene in the C57BL/6JCya strain of mice were knocked out. This region spans 860 bp of the coding sequence, representing 5.52% of the entire coding region. The gene knockout strategy is illustrated in Figure 1. After quality analysis of the sequences located 2000 bp upstream of Exon 4 and 2000 bp downstream of Exon 8, primers F1, F2, and R1 were selected for genotyping. The sequences were as follows:

USH2A‐F1: 5 ^′^‐TGGCAGGCTTTTACTTGAGCTATT‐3 ^′^;

USH2A‐F2: 5 ^′^‐GGGACGGGTAAATAGAAGTGACTA‐3 ^′^;

USH2A‐R1: 5 ^′^‐CAATGTGTGAGTCTGTCCATATC‐3 ^′^.

Genomic DNA was extracted from whole blood samples of wild‐type (WT), Ush2a^−/−^, and Heterozygous (Ush2a^+/−^) mice using the TIANamp Genomic DNA Kit (Tiangen Biotech, Beijing, China) according to the manufacturer′s instructions. Briefly, 100 *μ*L of blood was lysed in 500 *μ*L of lysis buffer, followed by incubation at 65°C for 30 min. After proteinase K digestion and isopropanol precipitation, DNA was resuspended in 50 *μ*L of nuclease‐free water. DNA concentration and purity were quantified using a NanoDrop 2000 spectrophotometer (Thermo Fisher Scientific, United States).

Three primer pairs (USH2A‐F1, USH2A‐F2, and USH2A‐R1) were designed to amplify distinct regions of the Ush2a gene. The primers were synthesized by Sangon Biotech (Shanghai, China). PCR amplification was performed in a 25‐*μ*L reaction volume containing 12.5 *μ*L of PrimeSTAR Max Premix (2×) (Takara, Japan), 1 *μ*L forward and 1 *μ*L reverse primers, 3.5 *μ*L of genomic DNA, and nuclease‐free water to final volume. The PCR program included an initial denaturation at 98°C for 10 s, followed by 40 cycles of denaturation at 98°C for 5 s, annealing at 55°C for 5 s, and extension at 72°C for 5 s, with a final extension at 72°C for 1 min.

PCR products were resolved on 1% agarose gels (prepared in 1× TAE buffer) stained with ethidium bromide (0.5 *μ*g/mL). Gels were run at 80–120 V for 30–40 min until the bromophenol blue dye migrated approximately 2 cm from the loading wells. Visualization was performed under a UV transilluminator (Bio‐Rad, United States), and gel images were captured using a Gel Doc XR+ system (Bio‐Rad, United States).

### 2.2. Auditory Brainstem Response (ABR) Testing

Mice were anesthetized via intraperitoneal injection of avertin (30 *μ*L/g), and the depth of anesthesia was confirmed using the righting reflex test, where mice were gently flipped onto their backs and observed for posture recovery within 1–2 min. Fully anesthetized mice were placed in a temperature‐controlled chamber (37°C) in the prone position. Sterilized electrodes (recording electrode at the midline of the skull, reference electrode posterior to the right pinna, and ground electrode posterior to the left pinna) were inserted after alcohol disinfection. A speaker was positioned 1–2 cm anterior to the mouse′s head to deliver acoustic stimuli. ABR testing was performed using BioSig software (Tucker‐Davis Technologies, United States) with the following parameters: click and tone burst stimuli (4–32 kHz) at sound pressure levels (SPL) ranging from 90 to 20 dB in 5‐dB decrements, with 256 repetitions per intensity level. Bandpass filters (100–3000 Hz) were applied to amplify waveforms. ABR waveforms were recorded, saved as digital files, and analyzed for thresholds (minimum sound intensity eliciting detectable waves) and latency/peak amplitudes of Waves I–V. Data were normalized to body weight and compared across WT, Ush2a^−/−^, and Ush2a^+/−^ groups to assess hearing function.

### 2.3. Immunofluorescence Staining

Mice were anesthetized with avertin (30 *μ*L/g) and decapitated. The cochlea was fixed in 4% PFA via round window perfusion for 24 h, decalcified in 0.2 M EDTA for 2–3 days, and rinsed in PBS. Under a stereomicroscope, the bony shell and connective tissues were removed to expose the organ of Corti. The basilar membrane was dissected into apical, middle, and basal turns, mounted on slides with the organ of Corti upward. After permeabilization with 0.2% Triton X‐100 (20 min) and PBS washes, phalloidin (0.5–1 *μ*g/mL) was applied to label stereocilia (30 min). DAPI (1 *μ*g/mL) was used for nuclear staining (10 min). Stained samples were imaged using fluorescence microscopy with filters for phalloidin (488/515 nm) and DAPI (358/461 nm) to analyze stereociliary and nuclear morphology across cochlear regions.

### 2.4. Hematoxylin and Eosin (H&E) Staining

Cochlear tissues were fixed and then longitudinally sectioned to 3‐mm thickness. After dehydration in graded alcohols (75%, 85%, 90%, 95%, and 100% ethanol for 4 h, 2 h, 1.5 h, 1.5 h, and 30 min, respectively), tissues were cleared in xylene (10 min × 2) and infiltrated with paraffin (1 h × 3). Paraffin‐embedded blocks were trimmed and sectioned. Dewaxed sections were rehydrated through xylene (20 min × 2), 100% ethanol (5 min × 2), and distilled water. H&E staining was performed sequentially: hematoxylin for 5 min, followed by differentiation in 85% ethanol and rapid eosin staining (5 s). Sections were dehydrated in 85% ethanol (5 min), 95% ethanol (5 min), 100% ethanol (5 min × 3), followed by clearing in butanol (5 min) and xylene (5 min × 2). Coverslips were applied with neutral resin after air‐drying. Morphological analysis was conducted under a light microscope.

### 2.5. Scanning Electron Microscopy (SEM)

Cochlear tissues were harvested from mice anesthetized with avertin (30 *μ*L/g) and fixed in SEM fixative (2.5% glutaraldehyde and 2% paraformaldehyde in 0.1 M PBS, pH 7.4) at 4°C for 24 h after perfusion through the round window under a stereomicroscope. The cochlear bony shell and spiral ligament were removed, followed by careful dissection to expose the organ of Corti. Tissues were rinsed three times in 0.1 M PBS (pH 7.4, 15 min each) and then postfixed in 1% osmium tetroxide in 0.1 M PBS (1 h) in a fume hood. After three additional PBS rinses, dehydration was performed in graded ethanol (30%, 50%, 70%, 90%, and 100% ethanol for 15 min each, with 100% ethanol repeated twice). Samples were critical‐point dried and sputter‐coated with a conductive layer for SEM imaging.

### 2.6. RT‐qPCR

Total RNA was extracted from cochlear tissues using Trizol reagent (Invitrogen) following standard protocols. RNA quality and concentration were assessed using a NanoDrop spectrophotometer (Thermo Fisher Scientific), with RNA integrity confirmed by agarose gel electrophoresis. Complementary DNA (cDNA) was synthesized from 1‐*μ*g RNA using the PrimeScript RT Master Mix (TAKARA, RR036A) with gDNA Remover to eliminate genomic DNA contamination. The reverse transcription reaction was performed at 37°C for 30 min, followed by 85°C for 1 min to inactivate the enzyme. Quantitative PCR (qPCR) was conducted using SYBR Green I‐based qPCR Premix (TAKARA, RR820A) on a LightCycler 96 system (Roche). Specific primer pairs for Fcer1g, Tgfb1, Irf7, Scn2a, Shank2, Bsn, and Prkce were designed with primer sequences validated for amplification efficiency (90%–110%) and specificity (spanning exon–exon junctions to avoid genomic DNA amplification). The reaction mixture (20 *μ*L) contained 10‐*μ*L 2× qPCR Premix, 0.4‐*μ*M forward and reverse primers, 2‐*μ*L cDNA template (diluted to 2.5 ng/*μ*L), and RNase‐free water. Amplification conditions included an initial denaturation at 95°C for 30 s, followed by 40 cycles of denaturation (95°C, 5 s), annealing/extension (60°C, 30 s), and fluorescence acquisition. A melting curve analysis (65°C–95°C, 0.5°C increments) was performed to confirm product specificity. Relative gene expression was normalized to the geometric mean of two stably expressed reference genes (*β*‐actin), validated using Genorm and NormFinder software. Data were analyzed using the 2^−*ΔΔ*Ct^ method. The primer sequences were as follows:

*β*‐Actin‐F: 5 ^′^‐GACTCCTATGTGGGTGACGAG‐3 ^′^; *β*‐actin‐R: 5 ^′^‐TCACGGTTGGCCTTAGGGTTC‐3 ^′^.

Bsn‐F: 5 ^′^‐ATTGTGTCCAGAAGGGCTCC‐3 ^′^; Bsn‐R: 5 ^′^‐GCCAGCTTGTAGATGCGTTA‐3 ^′^.

Fcer1g‐F: 5 ^′^‐CAGCCGTGATCTTGTTCTTGC‐3 ^′^; Fcer1g‐R: 5 ^′^‐TTCGGACCTGGATCTTGAGT‐3 ^′^.

Irf7‐F: 5 ^′^‐GCTCTGCCCACACAGGTTCT‐3 ^′^; Irf7‐R: 5 ^′^‐CATAGGGTTCCTCGTAAACACGGT‐3 ^′^.

Prkce‐F: 5 ^′^‐GCCACAGACGTTCCTTTTGG‐3 ^′^; Prkce‐R: 5 ^′^‐GGCTCCAGGTCAATCCAGTC‐3 ^′^.

Scn2a‐F: 5 ^′^‐GAGAGGATGCAGTGATCGCC‐3 ^′^; Scn2a‐R: 5 ^′^‐AAGAAGCGGAAGCTGTCAGG‐3 ^′^.

Shank2‐F: 5 ^′^‐CCCAGCTTATTCCAACCGCA‐3 ^′^; Shank2‐R: 5 ^′^‐TGTTGAGTGAAGGGGAACGG‐3 ^′^.

Tgfb1‐F: 5 ^′^‐ACTGGAGTTGTACGGCAGTG‐3 ^′^; Tgfb1‐R: GGGGCTGATCCCGTTGATT‐3 ^′^.

### 2.7. Transcriptome Sequencing

DEGs in cochlear tissues of Ush2a^−/−^ and WT mice were identified using the Lianchuan Biotech platform. Log2‐transformed fold changes (FCs) and *p* values were calculated to generate volcano plots and heatmaps, with FC thresholds set at |log2FC| ≥1 (corresponding to a 2‐FC) and *p* value thresholds at *p* < 0.05. The obtained DEGs were first classified into three categories—biological processes, cellular components, and molecular functions—via Gene Ontology (GO) analysis. Pathway enrichment analysis of DEGs was further performed using the Kyoto Encyclopedia of Genes and Genomes (KEGG) and Reactome databases to identify significantly enriched signaling pathways.

### 2.8. Statistical Analysis

All statistical analyses were performed using GraphPad Prism (Version 10.0, GraphPad Software, San Diego, California, United States). Data were presented as mean ± standard deviation (SD) for normally distributed variables. Comparisons between two groups were conducted using unpaired Student′s *t*‐test. For multiple group comparisons, one‐way analysis of variance (ANOVA) with Tukey′s post hoc test was applied. All statistical tests were two tailed, and significance was set at *p* < 0.05.

## 3. Results

### 3.1. Identification of USH2A Knockout Mice

**Figure 2:**
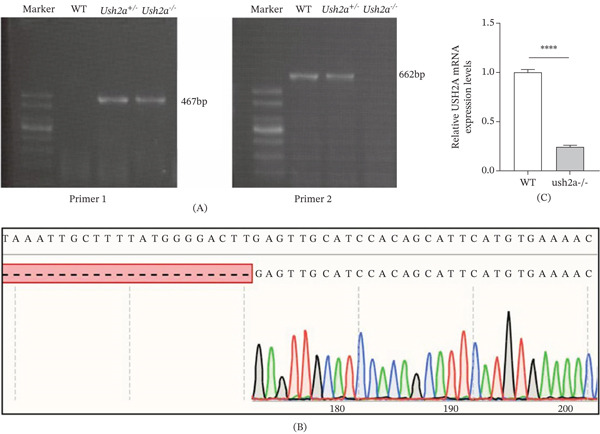
Identification of USH2A knockout mice. (A) PCR results analyzed by agarose gel electrophoresis. (B) Sanger sequencing was performed on Ush2a^−/−^ mice, and the sequencing results were aligned with the USH2A sequence from the NCBI gene database using SnapGene 6.0 software. (C) The USH2A levels in the WT and Ush2a^−/−^ mice were detected by RT‐qPCR assay.  ^*∗∗∗∗*^ *p* < 0.0001.

To identify the USH2A knockout mice, total DNA was extracted and subjected to PCR amplification, with PCR results analyzed by agarose gel electrophoresis (Figure 2A). WT mice exhibited a single 662‐bp band under Primer2 amplification, whereas Ush2a^−/−^ mice showed a single 467‐bp band under Primer1 amplification. Ush2a^+/−^ mice displayed two bands at 467 and 662 bp, respectively. Sanger sequencing was performed on Ush2a^−/−^ mice, and the sequencing results were aligned with the USH2A sequence from the NCBI gene database using SnapGene 6.0 software (Figure 2B), revealing sequence deletions in Ush2a^−/−^ mice. Finally, RNA was extracted from the cochlea of 3‐day‐old mice and analyzed by RT‐qPCR. As shown in Figure 2C, the USH2A mRNA levels in WT mice were significantly higher than those in USH2A knockout mice.

### 3.2. USH2A Knockout Mice Exhibited Reduced Hearing Sensitivity at Both Low and High Frequencies

**Figure 3:**
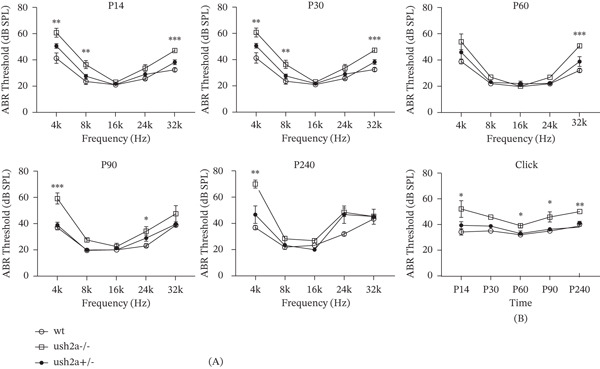
USH2A knockout mice exhibited reduced hearing sensitivity at both low and high frequencies. (A) The ABR testing was performed on WT, Ush2a^−/−^, and Ush2a^+/−^ mice at Postnatal Days 7 (P7), 14 (P14), 30 (P30), 60 (P60), 90 (P90), and 240 (P240). (B) Click‐evoked ABR thresholds of WT, Ush2a^−/−^, and Ush2a^+/−^ mice.  ^∗^ *p* < 0.05,  ^∗∗^ *p* < 0.01,  ^∗∗∗^ *p* < 0.001.

ABR testing was performed on WT, Ush2a^−/−^, and Ush2a^+/−^ mice at Postnatal Days 7 (P7), 14 (P14), 30 (P30), 60 (P60), 90 (P90), and 240 (P240) (Figure 3). Both Ush2a^−/−^ and Ush2a^+/−^ mice exhibited significantly reduced ABR thresholds at multiple time points, particularly at low frequencies (4 kHz) and high frequencies (32 kHz).

At P14, the numbers of WT, Ush2a^−/−^, and Ush2a^+/−^ mice were 4, 7, and 8, respectively. ABR thresholds in Ush2a^−/−^ and Ush2a^+/−^ mice were significantly lower than those in WT mice at 4, 8, and 32 kHz. At P30, the numbers were 4, 7, and 8, respectively. Similar significant reductions were observed in Ush2a^−/−^ and Ush2a^+/−^ mice at 4, 8, and 32 kHz. At P60, all three groups had five mice each. Ush2a^−/−^ and Ush2a^+/−^ mice showed significantly lower thresholds only at 32 kHz. At P90, the numbers were 5, 6, and 5, respectively. Significantly reduced thresholds in Ush2a^−/−^ and Ush2a^+/−^ mice were observed at 4 and 24 kHz. At P240, all three groups had three mice each. Only thresholds at 4 kHz were significantly lower in Ush2a^−/−^ and Ush2a^+/−^ mice compared to WT mice.

Click‐evoked ABR thresholds showed statistically significant differences at P14, P60, P90, and P240. Notably, within each group, ABR thresholds remained relatively stable over time. Compared to WT mice, Ush2a^−/−^ and Ush2a^+/−^ mice exhibited moderate‐to‐severe nonprogressive hearing loss, with more pronounced deficits at low (4 kHz) and high (32/24 kHz) frequencies.

### 3.3. The Modiolus, Stria Vascularis, and Basilar Membrane in USH2A Knockout Mice Were Found to Be Structurally Normal

**Figure 4:**
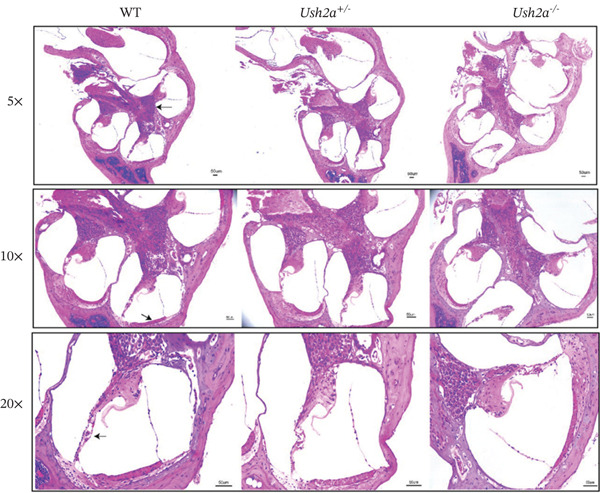
The modiolus, stria vascularis, and basilar membrane in USH2A knockout mice were found to be structurally normal. HE staining was used to examine the longitudinal sections of the cochlea in all groups at Postnatal Day 30. Scale = 50 *μ*m.

To evaluate the impact of USH2A knockout on the cochlear structure, HE staining was used to examine the longitudinal sections of the cochlea in all groups at Postnatal Day 30 (P30) (Figure 4). The modiolus, stria vascularis, and basilar membrane (indicated by black arrows) were observed under 5×, 10×, and 20× magnifications. No significant structural abnormalities were detected.

### 3.4. No Reduction in the Number of Inner and Outer Hair Cells (OHCs) Was Observed in USH2A Knockout Mice

**Figure 5:**
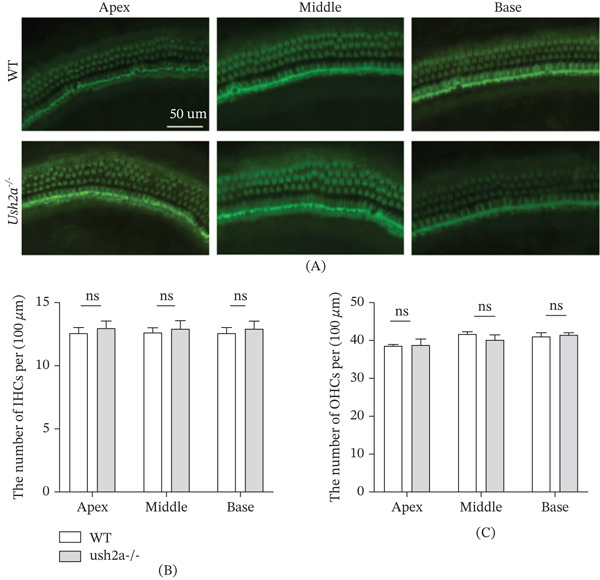
No reduction in the number of inner and outer hair cells was observed in USH2A knockout mice. (A) Immunofluorescence (phalloidin) staining was used to examine the cochleae of each group at Postnatal Day 30. The number of (B) IHCs and (C) OHCs in the basilar membrane at the apex, middle, and base of the cochlea was assessed.

To evaluate the impact of USH2A knockout on hair cell numbers, immunofluorescence (phalloidin) staining was used to examine the cochleae of each group at Postnatal Day 30 (P30) (Figure 5A). The number of inner hair cells (IHCs) and OHCs in the basilar membrane at the apex, middle, and base of the cochlea was assessed. Both the WT and Ush2a^−/−^ groups exhibited normal morphological characteristics of IHCs (Figure 5B) and OHCs (Figure 5C) at the apex, middle, and base. No statistically significant differences in the number of IHCs (Figure 5B) and OHCs (Figure 5C) were observed in USH2A knockout mice.

### 3.5. Morphological Changes of Cilia in OHCs of USH2A Knockout Mice

**Figure 6:**
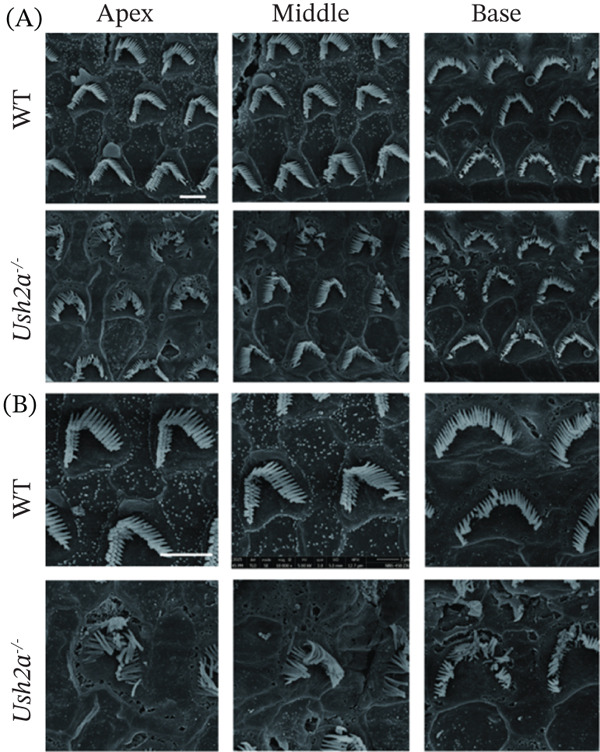
Morphological changes of cilia in outer hair cells of USH2A knockout mice. Scanning electron microscopy (SEM) was used to examine the stereocilia of hair cells in each group at Postnatal Day 30. (A) 5000× magnification. (B) 10,000× magnification.

To assess the impact of USH2A knockout on hair cell stereociliary morphology, SEM was used to examine the stereocilia of hair cells in each group at Postnatal Day 30 (P30) (Figure 6). At 5000× magnification, the stereocilia of OHCs in the WT group at the apex, middle, and base were neatly arranged. In contrast, the Ush2a^−/−^ group exhibited disorganized stereociliary bundles in some OHCs at the apex, middle, and base, with a higher proportion of irregular stereocilia observed at the apex (Figure 6A). At 10,000× magnification, severe stereociliary collapse was evident in the OHCs of the Ush2a^−/−^ group (Figure 6B).

### 3.6. Transcriptomic Analysis

**Figure 7:**
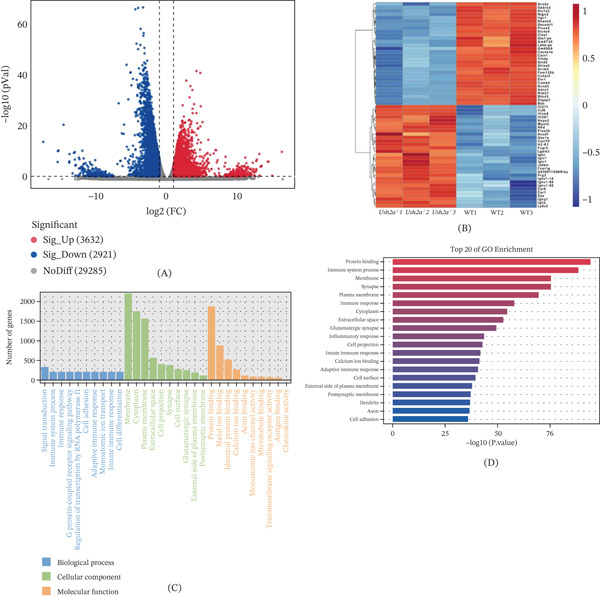
Transcriptomic analysis. DEGs in cochlear tissues of Ush2a^−/−^ and WT mice were identified using the Lianchuan Biotech platform and were calculated to generate (A) volcano plots and (B) heatmaps. (C) Functional enrichment analysis of DEGs was performed by categorizing them into three GO classifications. (D) Top 20 of GO enrichment.

Subsequently, we analyzed DEGs in the Ush2a^−/−^ and WT groups by transcriptome sequencing analysis. Compared to the WT group, the Ush2a^−/−^ group exhibited a total of 6553 DEGs, including 3632 upregulated genes and 2921 downregulated genes (Figure 7A). The fragments per kilobase of exon model per million mapped reads (FPKM) values of DEGs were log‐transformed and normalized using *Z*‐score analysis to generate a clustering heat map (Figure 7B). Functional enrichment analysis of DEGs was performed by categorizing them into three GO classifications: biological processes, cellular components, and molecular functions. The top 10 enriched terms in each category were shown (Figure 7C). Results showed that DEGs were primarily enriched in cellular components such as the cell membrane, cytoplasm, and cell membrane, participating in biological processes like signal transduction, immune system processes, and cell adhesion, and involving molecular functions such as protein binding, metal ion binding, and signal receptor activation (Figure 7D). Among these DEGs, the most DEGs associated with hearing loss were Scn2a, Shank2, Bsn, Fcer1g, Prkce, Tgfb1, and Irf7. Specifically, upregulated genes included Fcer1g, Tgfb1, and Irf7, whereas downregulated genes included Scn2a, Shank2, Bsn, and Prkce.

### 3.7. Enrichment Analysis of DEGs and Detection of Hearing Loss Associated Genes

**Figure 8:**
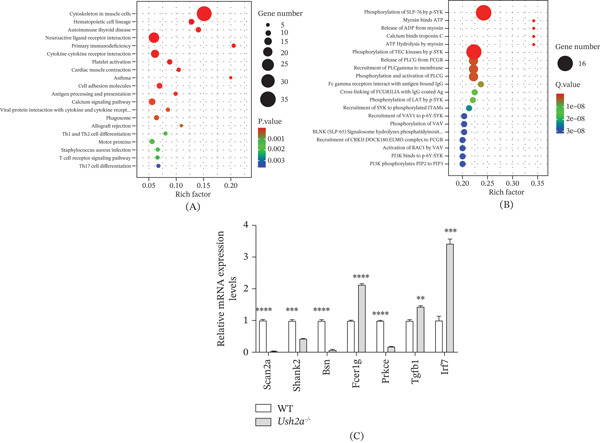
Enrichment analysis of DEGs and detection of hearing loss associated genes. (A) KEGG analysis of DEGs. (B) Reactome analysis of DEGs. (C) The mRNA levels of Scn2a, Shank2, Bsn, Prkce, Fcer1g, Tgfb1, and Irf7 in WT and Ush2a^−/−^ mice were detected by RT‐qPCR.  ^∗∗^ *p* < 0.01,  ^∗∗∗^ *p* < 0.001,  ^*∗∗∗∗*^ *p* < 0.0001.

Finally, using KEGG database, we enriched the pathways associated with DEGs and generated bubble plots. The 20 pathways with the smallest *p* values are shown in Figure 8A. The most significantly enriched pathway was cytoskeleton in muscle cells, primarily involved in cell motility. The motor protein pathway was also associated with cellular movement. In addition, using the Reactome database, we further enriched the pathways of DEG and generated bubble plots, selecting the 20 pathways with the smallest *Q* values (Figure 8B). Pathways related to cell motility included myosin binds ATP, release of ADP from myosin, and ATP hydrolysis by myosin. Finally, we validated the mRNA expression levels of Scn2a, Shank2, Bsn, Fcer1g, Prkce, Tgfb1, and Irf7 in mice. Compared to the WT group, the Ush2a^−/−^ group exhibited significantly reduced mRNA levels of Scn2a, Shank2, Bsn, and Prkce, whereas Fcer1g, Tgfb1, and Irf7 showed significantly increased mRNA expression (Figure 8C). These results were consistent with the transcriptomic analysis.

## 4. Discussion

USH is a leading cause of inherited deaf‐blindness, significantly impacting the quality of life for affected individuals. The most common form, Usher syndrome Type II (USH2), is primarily associated with mutations in the USH2A gene, which lead to sensorineural hearing loss and progressive vision loss due to retinitis pigmentosa [7, 8]. Approximately 85% of patients with USH2 have mutations in the USH2A gene, with notable variants such as c.2299delG being prevalent in the population [9]. Despite the identification of these mutations, the precise pathogenic mechanisms underlying USH2A‐related disorders remain poorly understood, necessitating further exploration into the genetic and molecular pathways involved in the disease.

The present study establishes a USH2A knockout mouse model to investigate the auditory consequences of USH2A gene disruption, revealing that Ush2a^−/−^ mice exhibit nonprogressive hearing loss across low‐ and high‐frequency ranges. Despite the absence of structural abnormalities in cochlear tissues (e.g., modiolus, stria vascularis, and basilar membrane) and preserved hair cell counts, significant morphological alterations in OHCs stereociliary bundles were observed, particularly at the cochlear apex. From this, it can be seen that the USH2A gene has no effect on the pathological structure of cochlear tissue, and its impact on hearing is related to changes in the morphology of OHCs.

OHCs are critical components of the cochlea in the inner ear, primarily located in the organ of Corti. Their primary physiological function is to enhance auditory sensitivity through electromechanical transduction [10]. OHCs not only detect vibrations induced by sound waves but also amplify acoustic signals via rapid length changes, a process termed the “cochlear amplifier” mechanism [11]. Studies have demonstrated that the mechanical movement of OHCs is closely linked to electrical signal transduction in the inner ear, as their activity influences signal transmission from IHCs, thereby modulating auditory perception [12]. Morphological alterations in OHCs are significantly associated with various types of hearing loss. For example, genetic studies have revealed that mutations in the STRC gene disrupt OHC morphology and function, leading to nonsyndromic hearing loss [13]. In contrast, noise‐induced hearing loss is strongly correlated with OHC morphological changes, with prolonged noise exposure causing OHC death and structural damage, particularly in high‐frequency regions [14]. Additionally, age‐related hearing loss is closely tied to progressive degeneration of OHC morphology and function, resulting in hearing decline [15]. These findings underscore that OHC morphological changes represent a key pathological feature of hearing loss and provide novel insights for classification and therapeutic strategies.

Previous research has identified USH2A localization at the synaptic regions of OHCs, where its spatiotemporal expression pattern contributes to synapse development [16]. This suggests that USH2A plays a vital role in OHC growth, morphogenesis, and functional regulation. Combined with our findings, we speculate that the auditory deficits observed in Ush2a^−/−^ mice arise from impaired OHC morphology. However, further studies are required to validate this hypothesis. For instance, advanced imaging techniques (e.g., super‐resolution microscopy) could quantify stereociliary bundle organization in Ush2a^−/−^ OHCs, whereas electrophysiological assays (e.g., compound action potential recordings) could assess cochlear amplification capacity. Additionally, comparative analysis of USH2A expression patterns in human USH patients and animal models may reveal conserved or species‐specific mechanisms underlying OHC dysfunction.

Transcriptomic analysis identified 6553 DEGs in Ush2a^−/−^ mice, with functional enrichment highlighting key biological processes such as signal transduction, immune response, and cell adhesion, as well as molecular functions like protein binding and metal ion transport. Notably, DEGs associated with hearing loss included Scn2a, Shank2, Bsn, and Prkce (downregulated) and Fcer1g, Tgfb1, and Irf7 (upregulated). These genes are critical for auditory signal processing, synaptic transmission, and cytoskeletal organization. For instance, Scn2a encodes a voltage‐gated sodium channel essential for action potential propagation in auditory neurons [17], whereas Shank2 and Bsn are scaffolding proteins involved in presynaptic glutamate release and synapse formation [18, 19]. The downregulation of these genes in Ush2a^−/−^ mice suggests a disruption in synaptic connectivity and neural coding of auditory signals. Conversely, upregulation of Fcer1g (a subunit of the high‐affinity IgE receptor) and Tgfb1 (a cytokine mediating inflammation and tissue remodeling) implies potential compensatory immune responses or pathological inflammation in the cochlea following USH2A loss [20, 21].

KEGG and Reactome pathway analyses revealed enrichment in cytoskeletal dynamics (e.g., “cytoskeleton in muscle cells” and “myosin binds ATP”) and motor protein activity, consistent with the observed stereociliary collapse in Ush2a^−/−^ OHCs. USH2A is known to interact with Usherin [22], a protein critical for linking actin filaments in stereocilia to the extracellular matrix [23], and its deletion likely destabilizes the actin‐based cytoskeleton, impairing mechanotransduction. The involvement of myosin‐related pathways further underscores the role of motor proteins in maintaining stereociliary polarity and fluid shear stress adaptation. These findings provide a molecular framework for understanding how USH2A mutations perturb hair bundle architecture and auditory function.

This study has several limitations. First, the sample size for longitudinal ABR testing was limited, particularly in older mice (e.g., P240), which may affect the generalizability of findings on age‐related hearing progression. Second, while transcriptomic data suggest candidate genes, functional validation (e.g., CRISPR‐based gene editing or pharmacological modulation) is required to confirm their roles in auditory pathology. Third, the absence of overt structural defects in IHCs and OHCs raises questions about the contribution of non–cell‐autonomous mechanisms (e.g., supporting cell dysfunction or neural degeneration) to hearing loss. Future studies should integrate single‐cell RNA sequencing to dissect cell‐type–specific gene expression changes and employ optogenetics to assess neural circuitry integrity in Ush2a^−/−^ mice. Additionally, translational research comparing USH2A variants in human patients with those in this mouse model could bridge the gap between basic science and clinical applications.

In conclusion, this study demonstrates that USH2A deficiency disrupts auditory function through stereociliary instability and dysregulation of genes critical for synaptic transmission and cytoskeletal dynamics. These findings advance our understanding of the molecular basis of USH and highlight potential therapeutic targets for preserving hearing in USH2A‐related disorders.

## Author Contributions

All authors participated in the design, interpretation of the studies and analysis of the data, and review of the manuscript. C.C. drafted the work and revised it critically for important intellectual content. B.X. and J.D. were responsible for the research design, whereas H.T. and P.B. shared the responsibility for data collection, survey, and data acquisition. Y.W. was responsible for data analysis and in‐depth interpretation of the collected data. Y.G. was responsible for revising and reviewing the manuscript.

## Funding

The study was supported by the National Natural Science Foundation of China Regional Program (Program Number: 82460222, 32160149, and 82360221).

## Disclosure

All authors read and approved the final manuscript.

## Ethics Statement

This study was approved by the Ethics Committee of Lanzhou University Second Hospital.

All animal experiments should comply with the ARRIVE guidelines.

All methods were carried out in accordance with relevant guidelines and regulations.

## Consent

The authors have nothing to report.

## Conflicts of Interest

The authors declare no conflicts of interest.

## Data Availability

The datasets used and/or analyzed during the current study are available from the corresponding author on reasonable request.
